# “Sandwich” mesh reconstruction of female giant urethral diverticulum: a case report

**DOI:** 10.1186/s12894-020-00598-2

**Published:** 2020-03-20

**Authors:** Juanjuan Xie, Bijun Liu, Jianjun Li, Zhigang Luo

**Affiliations:** Urology Department, The Second Hospital of University of South China, NO 35 Jiefangdadao, Henyang, China

**Keywords:** Urethra, Female, Diverticulum, Mesh, SUI, Reconstruction

## Abstract

**Background:**

There is no consensus between urologists on the diagnosis and treatment of female urethral diverticula. Once the diagnosis has been established, the most common treatment approach is surgical excision and reconstruction. Whether a staged procedure or simultaneous management is more appropriate for treating concomitant urethral diverticula and stress urinary incontinence remains controversial.

**Case presentation:**

A 63-year-old woman was hospitalized for repeated frequent urination, urgent urination, odynuria, and dysuria accompanied by intermittent overflow urinary incontinence for over 10 years. She had a 5 year history of urinary stress incontinence prior to onset of these symptoms and had had four urethral caruncles resected on four separate occasions. There was visible leakage of urine when abdominal pressure was increased during physical examination and urodynamic studies. Additionally, turbid urine was discharged when the anterior vaginal wall was squeezed. Cystourethrography showed circumferential filling with contrast and multiple bladder diverticulae in the mid plane of the pubic symphysis. Urethrocystoscopy showed an orifice to a diverticulum at 7 o’clock in the proximal urethra, into which an F19.8 urethroscope could be inserted, enabling examination of most of the diverticulae. The urethral diverticulae were resected, followed by mesh reconstruction of the urethra. During a 20-month follow-up, the treatment outcomes were satisfactory.

**Conclusion:**

We here report a case of a giant circumferential urethral diverticulum combined with stress urinary incontinence that was successfully managed by an uncommon surgical reconstructive technique: a minimally invasive “Sandwich” mesh repair procedure utilizing synthetic mesh wrap in the midurethral region.

## Background

The diagnosis and treatment of female urethral diverticula (UD) present a challenge to the urologist. Female urethral diverticula are considered relatively rare, with a prevalence of 1–6% [1]; however, their incidence is probably underestimated because they can be asymptomatic or misdiagnosed because of the characteristically nonspecific clinical manifestations such as vaginal mass, chronic pelvic pain, refractory lower urinary tract symptoms, and recurrent urinary tract infections [2]. However, increased awareness on physical examination and the use of imaging modalities such as magnetic resonance imaging have improved diagnostic accuracy [3].

Once the diagnosis has been established, the most common treatment approach is surgical excision and reconstruction [4]. When treating concomitant UD and stress urinary incontinence (SUI), some surgeons favor a staged procedure, whereas others recommend simultaneous management with an autologous pubovaginal fascial sling (APVS), which is reportedly a safe and effective means of managing this combination [5]. However, APVS is a lengthy invasive operation that may lead to additional complications.

Here, we report a woman with a giant circumferential UD and SUI who was treated by resection of the diverticulum and reconstruction of the urethra by a minimally invasive procedure incorporating use of synthetic mesh wrap in the midurethral region.

## Case presentation

**Fig. 1:**
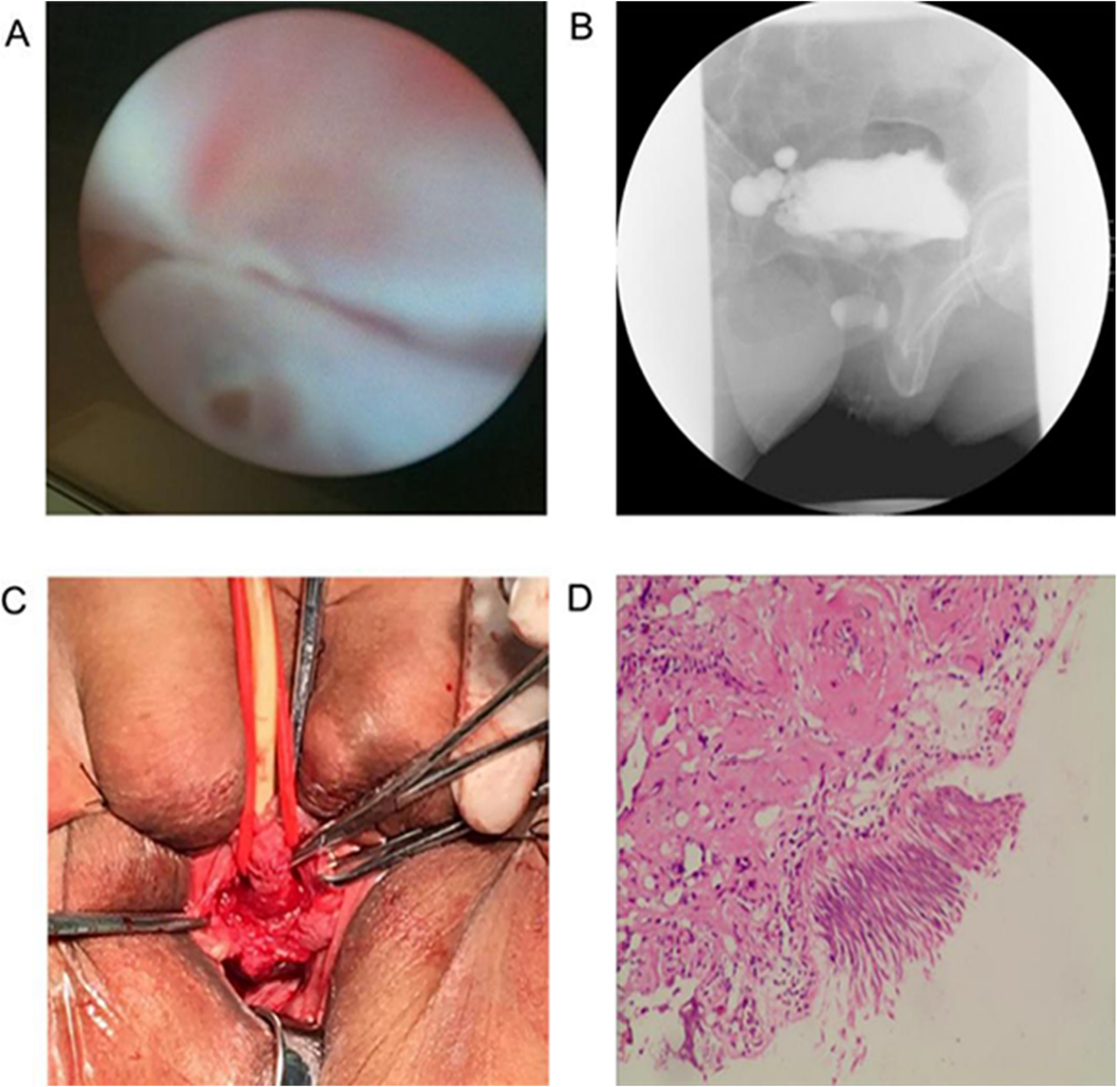
Evaluation, surgery, and pathology of a case of urethral diverticulum treated with an unusual method: the “Sandwich” mesh repair surgery. **a** Urethroscopy showed that there was a diverticulum orifice in the proximal urethra. The diverticulum could contain about 50 ml of physiological saline. **b** Cystourethrography showed the circumferential diverticulum around the urethra in the middle plane of the pubic symphysis. **c** After the diverticulum was removed, the urethra was fully free. The urethral circumference was used as a model to cut the Gynemesh mesh. **d** Urethral transitional epithelium was found at pathological examination. The diagnosis of urethral diverticulum was confirmed

A 63-year-old woman was admitted to the Second Hospital of University of South China for management of frequent urination, urgent urination, odynuria, and dysuria, accompanied by intermittent urine spillover for over 10 years. She reported that the greatest problem was that she had to lift her hips and change her posture constantly every time she urinated, passing some urine with each change in posture. She had a 5-year history of SUI before onset of these symptoms and had had four urethral caruncles resected 17, 16, 7 and 1 year before the current presentation. Additionally, she had not been sexually active for almost a decade. Physical examination showed an approximately 2.5 × 2.0 cm circular bright red polyp with a relatively wide base at the outer urethral orifice that bled when touched. In addition, turbid urine was passed when the anterior vaginal wall was squeezed. Urethroscopy showed the orifice of a diverticulum at 7 o’clock in the mid urethra. The diameter of the orifice was relatively large, enabling insertion of a F19.8 urethroscope into the diverticulum. The diverticulum cavity had smooth walls, and could be filled with 50 mL of normal saline (Fig. 1a). The normal course between the urethra and bladder was hard to identify. Extensive trabecular hyperplasia and multiple diverticula were found in the bladder. Cystourethrography confirmed multiple diverticula in the bladder, and a spherical area of contrast filling in the mid plane of the pubic symphysis (Fig. 1b). During cystourethrography, the patient passed only a small amount of urine, the residual urine volume being 300 mL. There was visible urethral leakage of urine when abdominal pressure was applied during the physical examination and urodynamic studies. A urodynamic study revealed an abdominal leak point pressure of 88 cm H_2_O, a maximal urethral closure pressure of 80 cm H_2_O, and a functional urethral length of 3 cm.

In accordance with findings on urine culture, piperacillin sodium and sulbactam sodium (2.5 g, q 12 h) were administered preoperatively and other preparatory measures were taken. The operation was performed by the chief urologist, who has over 30 years of experience, assisted by two other urologists. Epidural anesthesia was administered, after which then the patient was placed in the lithotomy position and a 20 F double-antrum air chamber catheter inserted into the bladder. A straight incision was made in the anterior vaginal wall. The vaginal wall and periurethral fascial were then dissected inferiorly and laterally along the wall of the diverticulum from the distal end of the urethral diverticulum to the pubocervical fascia and across the level of the bladder neck. When the diverticular sac was separated below the pubic symphysis, the whole diverticulum, which encircled the urethra, was completely exposed. Because there were extensive and advanced adhesions between the diverticulum and surrounding tissues, the wall of the diverticulum was resected in sections by dissecting it from the surface of the bulbospongiosus muscle. Next, the neck of the diverticulum and remaining diverticular tissue was resected, while preserving part of the diverticular neck. The remaining diverticular wall was varus sutured to the diverticular neck with 4–0 absorbable thread to restore urethral integrity and continuity, after which the sutured urethra was covered externally with the surrounding bulbospongiosus muscle and peri-urethral fascia and closed with absorbable sutures. The natural appearance of the urethra was restored as well as possible. Polypropylene mesh (10 × 15 cm; Gynecare Gynemesh) was tailored to serve as urethral mesh (1.5 × 3.0 cm) using the urethral circumference as a model and the mesh used to wrap 2/3 of the periurethral fascia of the mid-urethra and cover the neck of the diverticulum (Fig. 1c). Fixation was achieved by suturing the fascia and tissues surrounding the urethra, anchoring the mesh to the fascia and tissues between the urethra and the vaginal, in neither a retropubic nor transobturator manner, and then suturing the incision in the vaginal wall. Such three-layer suturing (periurethral fascia, mesh and vaginal wall flap) is known as “sandwich suturing”. Finally, the polyp at the urethral orifice was resected.

The volume of intraoperative blood loss was about 100 mL. The operation time was 105 min and the procedure was uneventful.

In accordance with the results of preoperative urine culture and drug sensitivity testing, piperacillin sodium and sulbactam sodium (2.5 g, q 8 h) were administered for 5 days. Pathological examination of the resected specimen showed transitional epithelial cells. Additionally, plasmocytes and lymphocytes were found infiltrating the area of the lesion (Fig. 1d). In addition, a polyp was found at the outer urethral orifice. The urethral catheter was removed 4 weeks after the procedure. Cystourethroscopy showed good recovery of the urethra with no mesh exposure.

The patient was followed up 6 and 20 months after the operation and reported that she no longer frequent urination, urgency, odynuria, dysuria, or incontinence. Routine urine examinations were negative. Ultrasound examination showed that a residual urine volume of about 10 mL. Her ICI-Q-SF score, using an internationally recognized incontinence scale, decreased from 12 preoperatively to 1 postoperatively.

## Discussion and conclusions

Approximately 10–60% of patients with UD also have SUI. In 10–33% of patients with UD, SUI is obscured by the influence of the mass, especially in patients with giant and proximal UD [6]. After surgery for an UD, about 10% of patients have SUI that subsequently requires a sling. Significant risk factors for the development of de novo SUI after diverticulectomy reportedly include a diverticulum larger than 30 mm and location in the proximal urethral [2, 7].

There is controversy on treatment options, including whether repair should be concomitant or staged. American Urological Association guidelines do not recommend use of synthetic mesh when performing concomitant repair because of the risk of complications such as erosion of the synthetic mesh, infection, and fistula formation, but rather recommend creation of an APVS [8, 9]. However, this is a lengthy and invasive procedure that may result in complications such as voiding dysfunction. There may also be problems with accessing sufficient fascial tissue. Some of the same considerations may apply when using synthetic mesh simultaneous with performing urethral diverticulectomy. However, others have reported that suburethral synthetic mesh tape can safely be used as sling material for treating SUI in patients with both SUI and UD, with no infection or exposure of synthetic mesh tape after a mean follow up of 33.3 months [10].

In the present patient, we performed mesh repair and reconstruction of the urethra after diverticulum resection, because: 1) the patient had a history of SUI and leakage of urine had been observed during physical examination and urodynamic studies; 2) the UD was greater than 3 cm in size and was located in the proximal and middle urethra; 3) because the patient had previously had four urethral caruncles resected, her urethra was short; 4) the diverticulum was so large that the periurethral fascia was attenuated and the intrinsic sphincter compromised; and 5) after we had informed the patient preoperatively that APVS might be more effective than mesh repair, and explained the complications of APVS and mesh repair, she chose mesh repair because it was the less invasive of the two procedures.

Use of midurethral synthetic mesh wrap to treat SUI coexisting with UD creates a certain degree of tension as a result of fixing the mesh to the fascia and tissues between the urethra and vagina while simultaneously providing support to minimize the risk of recurrence of a diverticulum at the point of the stalk of the transected diverticulum. To the best of our knowledge, simultaneous urethral diverticulectomy and suburethral wrap using synthetic mesh has not been reported previously. To decrease the complications of mesh repair and increase the success rate of the UD repair, we administered antibiotics both pre- and post-operatively. Additionally, to minimize the risk of mesh exposure, we fixed the mesh in place after covering the urethra with a layer of periurethral fascia and tissue and wrapping the mesh external to that. We followed our patient up for 20 months, during which there were no recurrences or complications and she expressed satisfaction with the treatment outcomes.

In conclusion, we here report a woman with a large urethral diverticulum presenting with SUI, which was successfully managed using an unusual surgical technique: “Sandwich” mesh repair surgery, a minimally invasive procedure incorporating use of midurethral synthetic mesh wrap. Because this report is of a single case, further and long-term studies are needed to confirm the effectiveness and safety of this approach.

## Data Availability

Data sharing is not applicable to this article as no datasets were generated or analysed during the current study.

## Abbreviations

UD: Urethral diverticulum
APVS: Autologos pubovaginal fascial sling
